# Sexual partner number and distribution over time affect long-term partner evaluation: evidence from 11 countries across 5 continents

**DOI:** 10.1038/s41598-025-12607-1

**Published:** 2025-07-31

**Authors:** Andrew G. Thomas, William Costello, Mons Bendixen, Leif Edward Ottesen Kennair, Menelaos Apostolou, Klára Bártová, Ondřej Burýšek, Rob Lowe, Peter Jonason, Marta Kowal, Yago Luksevicius de Moraes, Jiaqing O, Piotr Sorokowski, Danielle Sulikowski, Zuzana Štěrbová, Jaroslava Varella Valentova, Marco Antonio Correa Varella, Yan Wang, Arnaud Wisman, Paula Wright, Steve Stewart-Williams

**Affiliations:** 1 School of Psychology, Swansea University, Swansea, UK; 2 Department of Psychology, University of Texas at Austin, Austin, USA; 3 Department of Psychology, Norwegian University of Science and Technology, Trondheim, Norway; 4 Department of Social Sciences, University of Nicosia, Nicosia, Cyprus; 5 Department of Psychology and Life Sciences, Faculty of Humanities, Charles University, Pátkova, Czechia; 6 Psychology Research Institute, University of Economics and Human Sciences, Warsaw, Poland; 7 IDN Being Human Lab-Institute of Psychology, University of Wrocław, Wroclaw, Poland; 8 Department of Psychology, Faculty of Philosophy, Fundação Santo André, Santo André, Brazil; 9 Department of Experimental Psychology, Institute of Psychology, University of São Paulo, São Paulo, Brazil; 10 Department of Psychology, University of Macau, Macau, China; 11 Department of Psychology, Aberystwyth University, Aberystwyth, UK; 12 Institute of Psychology, University of Wrocław, Wrocław, Poland; 13 School of Psychology, Charles Sturt University, Wagga Wagga, Australia; 14 Department of Psychology, Faculty of Arts, Charles University, Prague, Czechia; 15 Department of Experimental Psychology, Institute of Psychology, University of Sao Paulo, Sao Paulo, Brazil; 16 Department of Psychology, Fudan University, Shanghai, China; 17 School of Psychology, University of Kent, Canterbury, UK; 18 Department of Life Sciences, Brunel University London, Uxbridge, UK; 19 School of Psychology, University of Nottingham Malaysia, Semenyih, Malaysia

**Keywords:** Evolutionary psychology, Cross-cultural psychology, Sex, Mate preferences, Sociosexuality, Sexual selection, Human behaviour

## Abstract

A prospective partner’s sexual history provides important information that can be used to minimise mating-related risks. Such information includes the number of past sexual partners, which has an inverse relationship with positive suitor evaluation. However, sexual encounters with new partners vary in frequency over time, providing an additional dimension of context not previously considered. Across three studies (*N* = 5,331) with 15 samples, we demonstrate that the impact of past partner number on a suitor’s desirability as a long-term partner varies as a function of distribution over time. Using graphical representations of a suitor’s sexual history, we found that past partner number effects were smaller when the frequency of new sexual encounters decreased over time. This moderation effect was stronger, and often curvilinear, when past partner numbers were higher. We replicated these findings in 11 countries from five world regions. Sex differences were minimal and inconsistent pointing to a lack of a sexual double standards. Sociosexuality (openness to casual sex) was a consistent moderator and tended to mute the sexual history effects. These findings suggest that people not only attend to a potential long-term mate’s quantity of sexual partners, but also the context surrounding these encounters such as pattern and timing. Together, the findings raise the possibility of an evolved mechanism for managing mating risks present in both sexes and across populations and adds nuance to a contentious topic of public interest.

**Supplementary Information:**

The online version contains supplementary material available at 10.1038/s41598-025-12607-1.

## Introduction

From “notches on the bedpost” in English to “Isoka” (a ladies’ man) in Zulu and from “Nikushoku-kei” (Carnivore-type) in Japanese to “rodada” (a woman with many ‘miles’) in Brazilian Portuguese, terms and phrases to describe sexual history and reputation appear common to the languages of the world. While these idioms can be used to demean or degrade, their omnipresence hints at the salience of sexual history in human mating psychology, possibly due to its evolutionary importance.

To understand why modern humans might draw attention to sexual history, we must consider the risks associated with sex and relationships in our ancestral past. These include the contraction of fertility-reducing sexually transmitted diseases (STDs)^1^; investing time, effort, and resources into someone who does not return in kind; intrasexual competition^2,3^ and sexual conflict^4^; for men, unknowingly investing in someone else’s offspring due to paternity uncertainty^5^; and, for women, being left ‘holding the baby’ due to asymmetries in obligatory parental investment^6^. Pleistocene humans, without access to the modern technologies (e.g., reliable contraceptives, family planning) and institutions (e.g., social safety nets) to mitigate some of these risks, needed to successfully navigate them or suffer fitness consequences.

Though a pair-bonding species, not all humans are strict, life-long monogamists. Humans possess both short- and long-term mating strategies^7^ and most engage in sexual exploration during adolescence, serial monogamy, or extrapair sex during their lifetime^8–10^. Individual differences in a prospective partner’s sexual history may therefore serve as an indicator of present or future risk in a long-term relationship. A suitor with an abundant sexual history may be more likely to have contracted an STD^11^, possess a strong desire for sexual variety that may motivate infidelity^12,13^, struggle to adhere to monogamous norms^14^, and have previous partners competing for their interests and willing to inflict costs on others^15,16^. Behavioural genetics evidence also reveals that individual differences in lifetime sexual partners^17^, sociosexuality^18^, and infidelity^19,20^ are all partially heritable; a reliable indication of individual intrinsic predispositions towards higher numbers of sexual partners.

While our small-community dwelling ancestors lacked the opportunities to have as many sexual partners as some of their modern city dwelling descendants, variability in partner number, though small, would have still been informative given the costs of poor mate choice. The benefits of tracking a potential partner’s sexual history, including their number of sexual partners and change in frequency of new sexual encounters over time, could have helped minimise risks and overcome some of the adaptive problems posed by long- and short-term mating^7^.

**Fig. 1:**
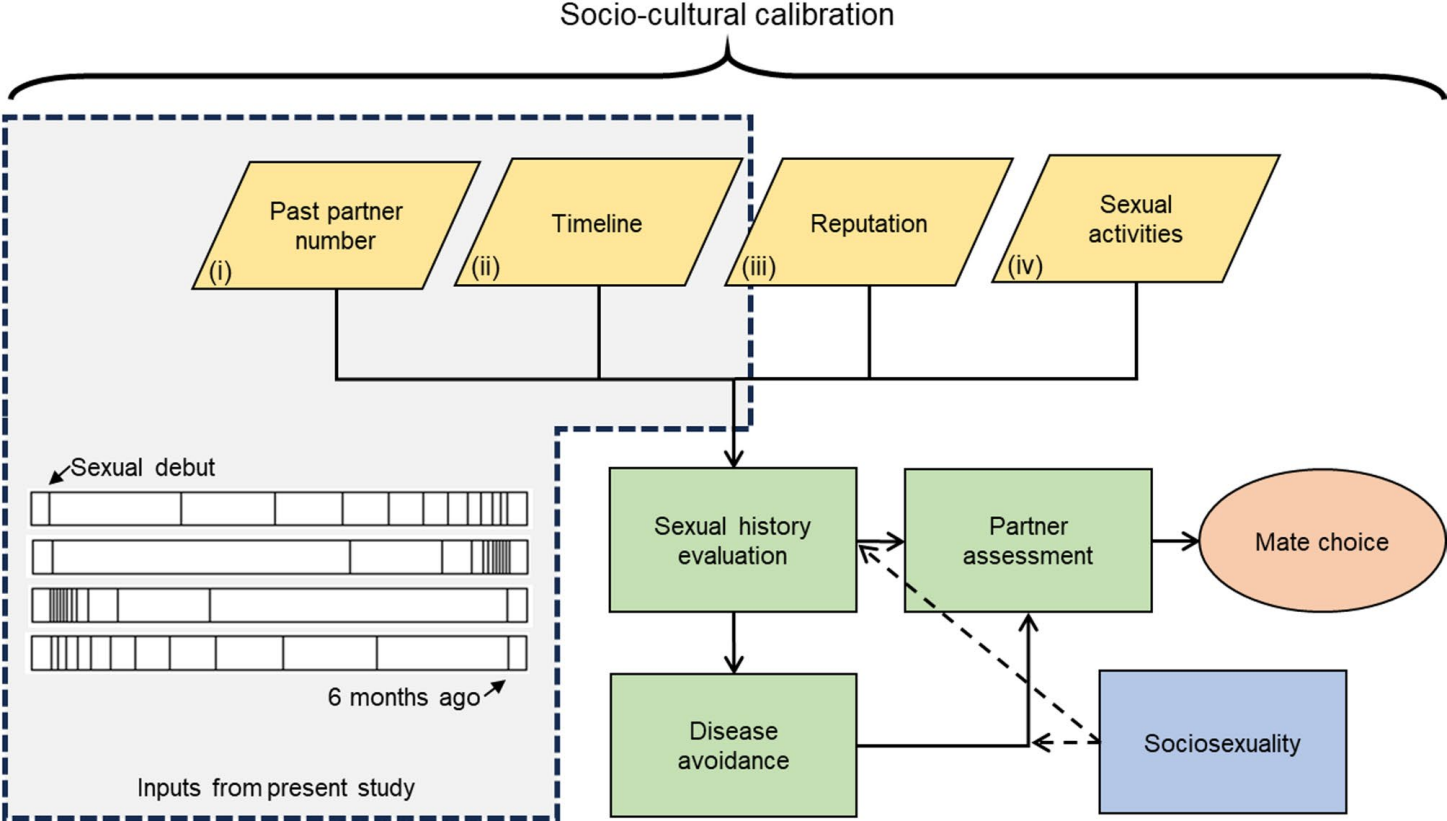
Past partner number and distribution of partners over time form part of a potential suitor’s sexual history which, in combination with disease avoidance mechanisms, informs partner assessment and subsequent mate choice—an assessment moderated by mating strategy (sociosexuality).

We hypothesise that humans who paid mind to the sexual history of others and used this to guide decision making in mating would have minimised mating risks, enhanced their fitness over others and, consequently, passed this tendency to future generations. This process has brought us to the point where our modern-day mating psychology attends to and evaluates the sexual history of others, through inputs such as (i) past partner number (PPN)^21^, (ii) the timing and circumstances of sexual relationships, (iii) sexual reputation^22^, and (iv) types of sexual activities^23^ (see Fig. 1). Sexual history evaluation then informs partner assessment and subsequent mate choice, working in conjunction with disease avoidance mechanisms^24^ and, much like disease avoidance, moderated by sociosexuality (openness to casual sex) to facilitate short-term mating^25,26^.

The role of sexual history and its impact on partner appraisal and mate choice have been considered before, particularly in Western countries. Such research revealed that willingness to get involved with (e.g., date) a suitor declines as their number of past sexual partners increases^27,28^, with few sex differences^29^, and a slightly more muted effect in short-term mating contexts^21^ or among those employing a short-term mating strategy^28^. Research also points to the existence of curvilinear relationships such that some sexual history is preferable to no sexual experience at all, at least in the United Kingdom^21^. Other research has focused on the presence or absence of sexual double standards, generally finding that liberal sexual reputation and activity leads to harsher judgements^23,29,30^ but providing little evidence that the sexual history of men is evaluated differently to that of women^31,32^.

From a cross-cultural perspective, variation in the importance of chastity is less consistent between cultures than traits like good health or kindness^33–35^. However, finding the absence of previous sexual activity to be relatively unimportant does not imply the absence of processing information associated with previous sexual activity. We therefore seek to develop deeper insight into cross-cultural variation in how sexual history is processed and used to inform mate choice.

Adopting an adaptationist perspective to investigate the role of sexual history within mating psychology puts mating risk at theoretical front-and-centre. Reliable and partially heritable indicators of risk become key inputs to guide long- and short-term mating decisions. This perspective can illuminate previously overlooked design features of, and inputs for, the psychological mechanisms that evaluate sexual history. One such input is temporal distribution – that is, how past sexual partners are spread across time (Fig. 1, ii). Consider two individuals with 12 past partners. For Person A, most of their new sexual encounters happened near the time of their sexual debut and have reduced in frequency over time, while Person B had the opposite experience; they had few new partners near their sexual debut and these increased in frequency over time (bottom-left, Fig. 1). Choosing Person A as a potential long-term partner might pose less of a risk compared to B because (a) the outward symptoms of STDs may have had more opportunity to manifest themselves, (b) they may no longer be in a period of sexual experimentation and be more willing to commit to a partner, lowering intersexual conflict, and (c) previous partners are less likely to be present, reducing potential intrasexual competition. Thus, as frequency decreases and risk lowers, so might we expect the negative impact of high past partner numbers to diminish - an attenuation effect. There have only been limited attempts to investigate such effects, with extant research focusing on duration of^36^, or time since^37^, a suitor’s last relationship or declarations of a change in relationship preference^38^. A key aim of this research is to provide a comprehensive investigation of the role of partner frequency change over time.

In the current work, we examine the effects of two pieces of sexual history information on partner appraisal: past partner number and frequency change over time. While this information is likely used as part of both long- and short-term mate evaluation, we focus on the former here. We predict that one’s willingness to consider a long-term relationship with someone will be negatively related to their number of past sexual partners (*Prediction 1*) and an increased frequency of new sexual encounters (*Prediction 2*). As the effect of frequency change may be more prominent at higher levels of past partners, we also predict their interaction (*Prediction 3*).

The risks associated with poor mate choice are more sexually symmetrical in long-term mating contexts than short-term ones because both sexes invest heavily in the relationship and have high levels of parental investment^6^. That is not to say that no sex differences exist; women are more susceptible to STDs^26^ and more likely to be killed at the hands of their partners than men^39^. Nonetheless, a converging long-term mating psychology between the sexes, driven by shared adaptive problems and risks, leads us to predict minimal sex differences among our three predicted effects (*Prediction 4*). Like other aspects of our evolved mating psychology^33^, we expect sexual history evaluation mechanisms to be highly canalised. That is, we expect cross-cultural variation in the strength of past sexual partner number and frequency change effects to be a matter of degree and not kind. The effects themselves should be present in all cultures (*Prediction 5*). Finally, because disease avoidance mechanisms are suppressed by short-term mating strategy, we expect that sociosexuality will moderate the impact of past partner number and frequency change (*Prediction 6*).

## Results

### Past partner number effects

**Fig. 2:**
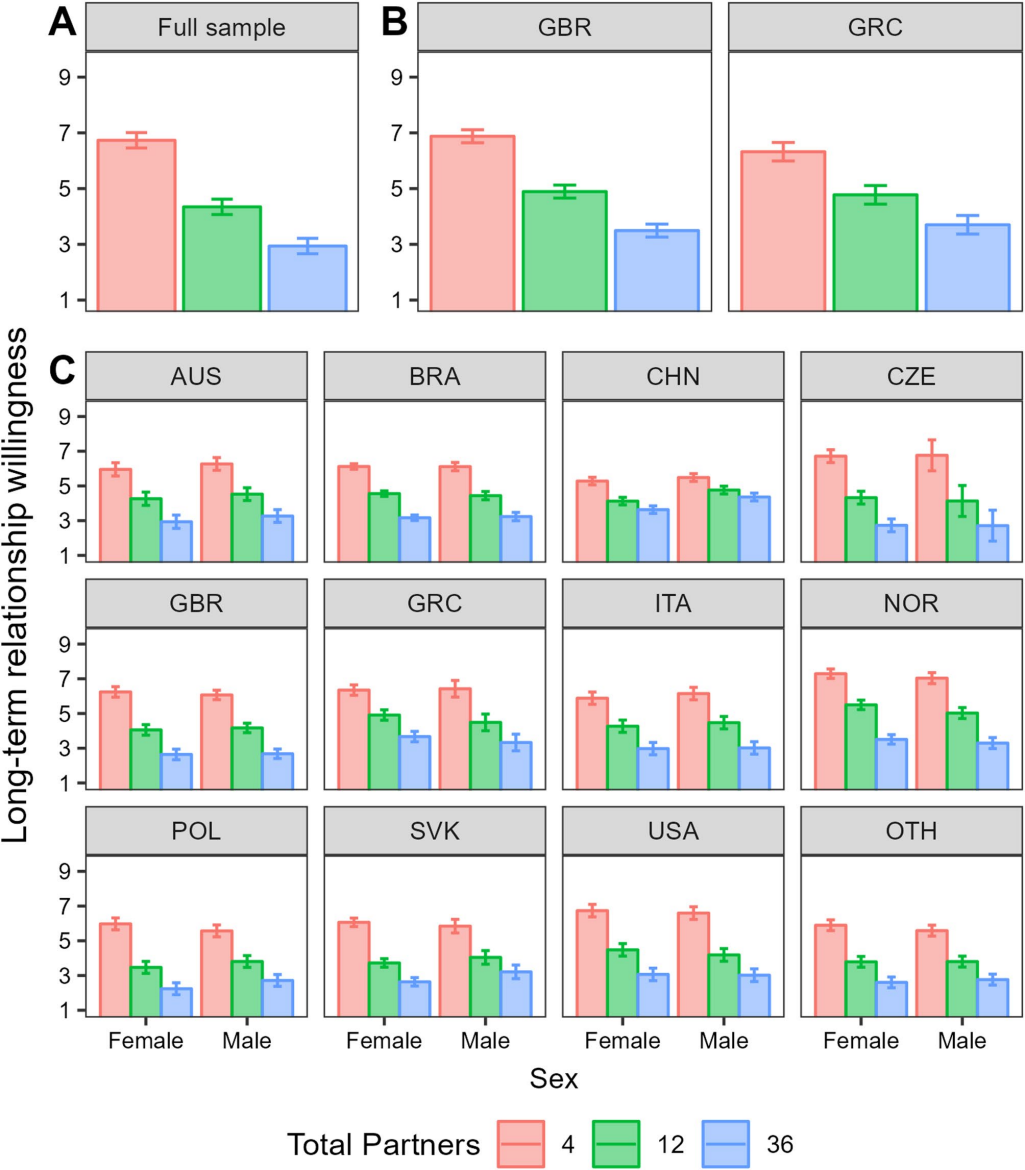
The effect of past partner number on willingness to enter a long-term relationship with a prospective partner. Panels (**A**–**C**) show results from studies 1 to 3 respectively. Bars reflect estimated marginal means with 95% CIs. Abbreviations reflect ISO 3166-1 alpha-3 country codes.

We began by modelling the effect of past partner number collapsed across distribution. As can be seen in Fig. 2, across all three studies there was a robust effect of past partner number on willingness to consider a long-term relationship.

#### Study 1

In S1, there was a large effect of Past Partner Number (*p* < .001; η_p_^2^ = 0.60) with decreases in willingness between 4 and 12 past partners (*p* < .001; *d* = 1.13) and 12 and 36 past partners (*p* < .001; *d* = 0.67). There was neither a main effect of sex nor a two-way interaction.

#### Study 2

In S2, the large effect of PPN was replicated (*p* < .001; η_p_^2^ = 0.54) but there was also an interaction between PPN and country (*p* < .001; η_p_^2^ = 0.02). In Greece, there were clear decreases in willingness from 4 to 12 (*p* < .001, *d* = 0.79) and 12 to 36 (*p* < .001, *d* = 0.55) past partners. These effects were similar but slightly larger in the UK (*p* < .001, *d* = 1.02 and *p* < .001, 0.72, respectively). There was neither a fixed effect of sex nor an interaction involving sex.

#### Study 3

Finally, S3 replicated the large effect for a third time (*p* < .001; η_p_^2^ = 0.50). This was qualified by a three-way interaction between PPN, country, and sex (*p* < .001; η_p_^2^ < 0.01). In all countries, willingness decreased between 4 and 12 past partners, ranging in size from *d* = 0.46 (in China) to 1.24 (in Czechia). Between 12 and 36 past partners, the decrease ranged in size from *d* = 0.22 (in China) to 0.92 (in Norway). After applying Type I error corrections, the only significant sex differences were found in the Chinese sample, where men showed greater willingness than women in the 12 (*M* = 4.76, *SE* = 0.11 vs. *M* = 4.12, *SE* = 0.11, *p* < .001) and 36 (*M* = 4.36, *SE* = 0.11 vs. *M* = 3.63, *SE* = 0.11, *p* < .001) past partner conditions. Both sex differences were small-to-medium in size (*d* = 0.31 and 0.36).

#### Summary of past partner number effects

Across all studies, there was a clear effect of PPN. The weighted average effect size for the decrease in willingness between 4 and 12 partners was large (*d* = 0.87, *SE* = 0.01), and medium in the case of 12 and 36 partners (*d* = 0.59, *SE* = 0.01). Differences between countries were confined to effect size, but never its presence or direction. Sex differences were minimal, but when present, showed no evidence that the sexual history of a suitor was judged more harshly by men than by women. Models and follow-up analyses can be found in Tables S1-S3.

### Distribution

**Fig. 3:**
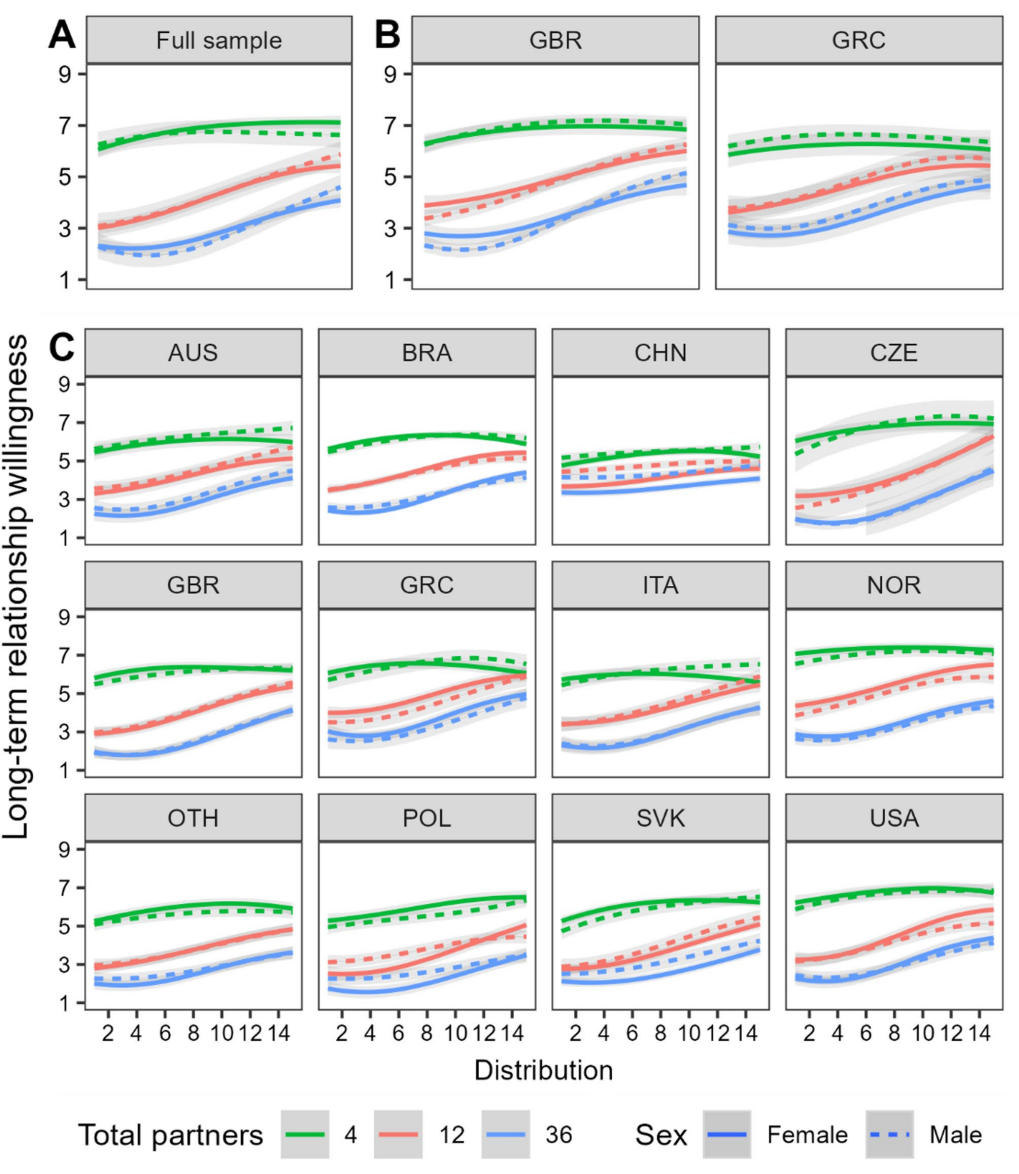
The effect of past partner number and frequency distribution on willingness to enter a long-term relationship. Panels (**A**–**C**) show results from studies 1 to 3 respectively. Distributions range from a sharp increase in frequency of new partners over time (1) to equally spaced across time (8) to a sharp decrease in frequency over time (15). Ribbons reflect 95% CIs. Abbreviations reflect ISO 3166-1 alpha-3 country codes.

Next, we considered how distribution affects willingness and whether this interacts with past partner number. As can be seen in Fig. 3, all three studies showed a robust effect of distribution on willingness. In general, willingness was highest when the frequency of new sexual partners decreased over time and lowest when the frequency increased over time.

#### Study 1

In S1, the main effect of distribution (*p* < .001, η_p_^2^ = 0.10) was qualified by a three-way interaction between distribution, PPN, and sex (*p* < .001, η_p_^2^ < 0.01). In all PPN conditions, willingness ratings were higher for distributions showing a marked decrease in the frequency of new partners over time. For 4 partners, the increase from one end of the distribution to the other was small (*d* = 0.31, *p* < .001) and quadratic in nature (*z* = 4.80, *p* < .001). For both 12 and 36 partners, the change was large (*d* = 1.10, *p* < .001 and *d* = 0.85, *p* < .001) and cubic (*z* = 2.87 and 4.85, both *p*s < 0.01). The cubic relationship suggests that the distribution effect suffers from inertia at one extreme and diminished returns at the other. Sex differences were constrained to the distributions showing decreased frequency of new partners. At their peak, sex differences were small (*d* = 0.20–0.22) and not statistically significant (*p*s > 0.077).

#### Study 2

In S2, we replicated the main effect of distribution (*p* < .001, η_p_^2^ = 0.11). We also found a two-way interaction between distribution and PPN (*p* < .001, η_p_^2^ = 0.04), but the three-way interaction involving sex was only marginally significant (*p* = .06, η_p_^2^ < 0.01). No other interaction involving both PPN and distribution was present. There was a three-way interaction between distribution, country, and sex (*p* < .001, η_p_^2^ < 0.01). The general pattern was that in all past partner conditions, willingness increased as the frequency of new partners showed a reduction over time. For 4 partners, the increase from one end of the distribution to the other was small (*d* = 0.20, *p* < .001) and quadratic in nature (*z* = 6.73, *p* < .001). For both 12 and 36 partners, the change was large (*d* = 0.96, *p* < .001 and *d* = 0.89, *p* < .001) and cubic (*z* = 4.81 and 7.67, both *p*s < 0.001). As in S1, sex differences were constrained to the distributions showing decreased frequency of new partners. Women showed greater willingness than men in the 36-partner condition, though even the largest sex difference (distribution point 13) was only *d* = 0.16 and non-significant after Type I error adjustment. Collapsing across PPN conditions, the effect of frequency distribution on willingness showed a stronger linear trend for women than men in the British (z = 4.385, *p* < .001) but not the Greek (z = 0.862, *p* = .389) sample.

#### Study 3

Here, there was a main effect of distribution (*p* < .001, η_p_^2^ = 0.07) qualified by a four-way interaction between distribution, PPN, country, and sex (*p* = .001, η_p_^2^ < 0.01). In all countries and PPN conditions, willingness increased as the frequency of new sexual encounters decreased over time. For 4 partners, the increase from one extreme of the frequency distributions to the other had an effect size ranging from *d* = 0.15 (Norway) to *d* = 0.60 (Slovakia). For 12 partners it ranged from *d* = 0.31 (China) to *d* = 1.50 (Czechia), and for 36 partners it ranged from *d* = 0.29 (China) to *d* = 1.10 (Czechia). The relationship between distribution and willingness was generally curvilinear in some way. For 4 past partners, all countries apart from Poland showed a quadratic relationship. At 12 partners, linear relationships were present only for Australia, China and the ‘Other’ sample, the rest were curvilinear with a cubic relationship (*n* = 7) being most common and for 36 past partners every country showed a curvilinear pattern with the most common being cubic (*n* = 8).

The role of sex in the interaction term reflected a small number of modest differences in the decreasing frequency distributions. When responding to the distributions showing the sharpest decrease over time, Italian men were more willing to get involved with someone who had 4 past partners than Italian women were (*d* = 0.41, *p* = .006), while the sex differences were reversed in the case of Norwegian men and women in the 12-partner condition (*d* = 0.28, *p* = .038). The only other notable sex differences occurred in the Chinese sample where men were generally more favourable towards a suitor than women at both distribution extremes. Sex differences were present for both 12- (*d* = 0.33, *p* < .001) and 36-partner (*d* = 0.34, *p* < .001) conditions when the distribution showed the sharpest increase in frequency and present for the 4- (*d* = 0.22, *p* = .021) and 36-partner (*d* = 0.30, *p* < .001) conditions when the distribution showed the sharpest decrease in frequency.

#### Summary of distribution effects

Across all studies, distribution consistently affected perceptions of a potential suitor with willingness being highest when the distributions showed a sharp decrease in new partners over time, and lowest when they showed a sharp increase over time. This effect was small (weighted average *d* = 0.28, *SE* = 0.01) and followed a quadratic pattern for 4 past partners, while it was much larger (weighted averages *d* = 0.84, *SE* = 0.01 and *d* = 0.72, *SE* = 0.01, respectively) and cubic for 12 and 36 past partners.

The curvilinear patterns suggest the presence of inertia and diminished returns effects. When past partner numbers are low, shifts towards a pattern of reduced frequency of new partners has an immediate, positive impact on willingness which soon suffers from diminished returns. These diminished returns are also present with higher numbers of past partners. Here we also see an inertia effect – a clearer movement of sexual history toward reduced frequency is required before subsequent effects on willingness are seen. The combination of inertia and diminished returns leads to a cubic pattern most clear in the 36-partner condition. As previously found, differences between countries were confined to the size of the effect and the extent of its curvilinear nature, but never its presence or direction. Sex differences were minimal and showed no evidence of a sexual double standard. Models and follow-up analyses can be found in Tables S4-S6.

### Sociosexuality

**Fig. 4:**
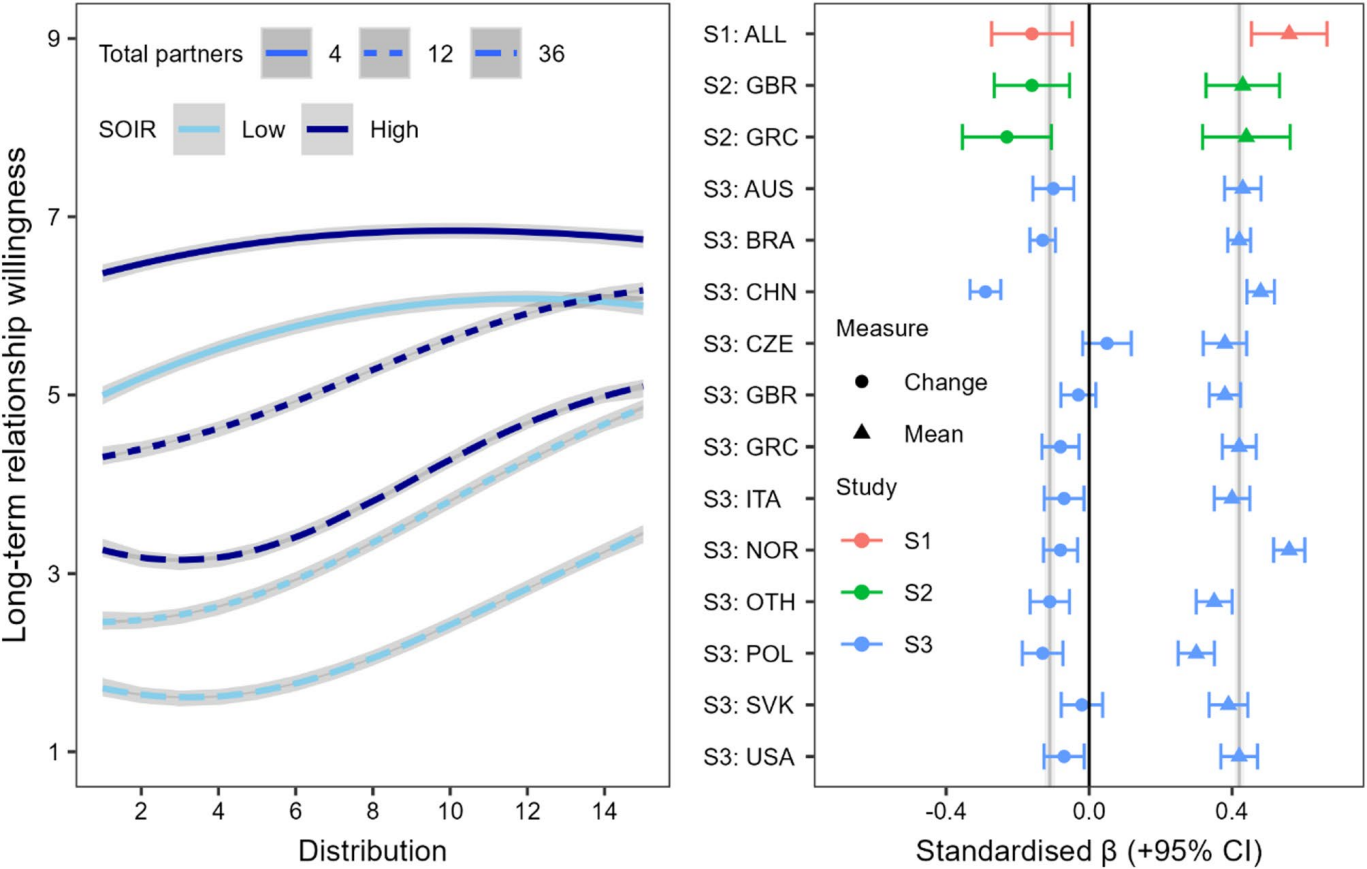
Impact of sociosexuality on past partner number and distribution effects. The left panel compares high (+ 1 SD) and low (− 1 SD) groups in S3 with 95% CI. The right panel shows standardized effects of sociosexuality on strength of the frequency distribution effect (change) and willingness collapsed across distribution (mean) for the 12 past partner condition in all studies. Grey lines represent weighted averages and 95% CI. Abbreviations reflect ISO 3166-1 alpha-3 country codes.

As seen in Fig. 4, unrestricted sociosexuality lessened the impact of past partner number and frequency distribution on willingness, with a 4-way interaction between PPN, distribution, Sociosexuality (SO), and country (*p* < .001, η_p_^2^ < 0.01). When moving from the ‘increased frequency’ end to ‘reduced frequency’ end of the distribution, those higher in SO (+ 1 *SD*) showed smaller increases in willingness than those lower in SO (-1 *SD*) for both 4 (*d* = 0.18 vs. *d* = 0.46) and 12 (*d* = 0.84 vs. *d* = 1.08) past partners, but not 36 (*d* = 0.81 vs. *d* = 0.78). This likely reflects a ceiling effect among those with more unrestricted sociosexuality when a prospective partner has a small or medium number of past partners.

When considering the 12 past partner condition only, we find a consistent, replicated pattern across all studies and samples: unrestricted sociosexuality was associated with greater willingness to get involved overall (weighted average β = 0.42, *SE* = 0.01). We also find a reduction of the distribution effect (weighted average β = -0.11, *SE* = 0.01) in 80% of the samples.

These findings suggest that because the effect of partner number is smaller among those high in SO, the effect of distribution has a less pronounced impact on their assessment of potential partners. Importantly, however, the presence of these two effects is consistent: Even those with an unrestricted sociosexuality show reduced willingness when numbers of sexual partners are large and increasing in frequency. Models and follow-up analyses can be found in Tables S7-S8.

## Discussion

We found strong evidence that modern humans are sensitive to the sexual history of potential partners and show a clear preference for fewer past partners. Across 15 sub-samples, there was a very large effect of past partner number, with the 13 country-specific sub-samples not only confirming the findings of earlier work^21,27–29^ but revealing that this effect is present not only in European and North American countries but also in South American, Asian, and Australasian ones as well.

We also found that frequency distribution matters when appraising a suitor’s sexual history. If a suitor’s new sexual encounters became less frequent over time, they were rated more favourably than someone who had new partners regularly or were increasing the frequency of new partners over time. The size of this effect was notably smaller than the effect of past partner number but it was still medium-to-large in size. The interaction between past partner number and frequency distribution demonstrated that the impact of distribution was greater when the number of total past partners was larger. In some cases, and particularly in the highest past partner number condition (36 partners), the effect of distribution was curvilinear. This reveals the presence of inertia and diminished returns with respect to changes in frequency. While an increase in frequency negatively impacted willingness, beyond a certain threshold, further increases had little additional effect. Decreases in frequency showed a similar, though less consistent, threshold effect.

When examining *societal* attitudes to sexual behaviour, a pattern of greater scrutiny of female sexual history appears^31,32^. However, research on *personal* attitudes tends to reveal a lack of sexual double standards^23,29,30^ and the current work is no exception. Sex differences tended to be minimal, small-to-medium in size, and inconsistent across countries. This supported our predictions and makes theoretical sense given that sex differences in the risks associated with sex and relationships would have been smaller (but not identical) for ancestral men and women in long-term mating contexts compared to short-term ones^6^. In theory, the differences may be larger and more consistent across samples within the context of short-term mating because some of the mutual risks of long-term mating (e.g., infidelity and lost investment) become redundant in this context, leading to a more sexually asymmetrical risk profile.

The fixed effect of country and the interaction effects including country were consistently small. Nonetheless, cultural variation existed. Considering 4 past partners, for example, participants in some countries (e.g. Norway, USA) tended to show more willingness to get involved with a suitor than others (e.g., Poland, China). These differences might be explained by sociocultural differences in sexual liberalism^40–42^, or variation in ecological variables like sex ratio^43^ and environmental demand^44^. For example, even after the onset of a sexual revolution in China^45^, it still scores much lower on sexual liberation compared to Western countries^46^, And in terms of sex ratio, Chinese men and women currently 21 years old were born in a year (2004) where there were 1.2 men for every 1 woman^47^ compared to 1.02 to 1 in Norway^48^. Larger studies of this phenomenon with more countries and regional representation would allow the influence of country-level predictors to be examined.

While there is value in explaining cultural variability in sexual history evaluation, our original prediction was related to cultural invariance and so we will refrain from being overly speculative about variation between countries. Instead, focusing on the forest rather than the trees reveals remarkable consistency in the effects of past partners and frequency distributions, suggesting that the use of these inputs in partner assessment is, much like other aspects of our mating psychology, highly canalised^33^.

One variable that accounted for more variability in past-partner effects than country of upbringing was sociosexuality. Sociosexuality, often used as a proxy for mating strategy^49^, has a moderating effect on some aspects of mating psychology, particularly those that might act as barriers to successful short-term mating^25^. In the current study, those with a more unrestricted sociosexuality, were less influenced by past partner number and slightly less sensitive to frequency change. Nonetheless, both these effects were still present showing that even those with a short-term mating strategy attend to the risks associated with sex and relationships.

The current study is strengthened by its efforts to (i) replicate the central effects across several samples from a diverse array of countries using a relatively large sample of individuals and (ii) sophisticated analyses which allowed for the examination of curvilinear relationships. It is also strengthened by its real-world relevance. The topic of “body count” attracts a great deal of discussion online. Even a superficial search of YouTube with terms like “body count sex” reveals thousands of videos on the topic, often including denigrating discourse promoting sexual double standards. (As just one example, a recent video clip from the “@whatever” podcast entitled “They just CAN NOT ACCEPT men prefer low body count women?! ” has received over 1 million views in three months^50^). Our scientific findings, which show a lack of sexual double standards and that context is important when considering sexual history, adds a nuanced view to an issue of great public interest that could counteract inaccuracies in the public discussion of sexual history, potentially reducing sexist discourse online.

At the same time, the study has limitations. These include a lack of representative samples from within each country, and the use of pictorial representation to examine the effects of frequency change. While providing greater control within the study, these stimuli are divorced from the way sexual history information is conveyed in social settings, such as through gossip and story^22,51^. Lastly, there is a debate about to what extent self-reported preferences inform actual mate choice^52^ that raises the possibility that past partner and frequency distribution effects have little influence on partner selection. Many of these limitations point to possible future directions of this work. These include the aforementioned deeper examination of cross-cultural variance in these sexual history effects and the integration of sexual history into wider mating preference work that, despite capturing participant preferences across a large number of traits, neglects to include sexual history^33,53^. In exploring the former, it is worth including cultures that vary in mating system. The countries in the current study legally enforce monogamy. However, human mating psychology evolved in the context of low levels of polygamy^54^. As such, it’s worth considering how sexual history evaluation might differ in countries which permit polygyny and polyandry. In exploring the latter, a full understanding of how past partner number and frequency are used to inform mate choice can only be achieved by considering both short- and long-term mating contexts. Indeed, using Sexual Strategies Theory^7^ we would predict greater sex differences when evaluating the sexual history of a suitor in a short-term context compared to a long-term one. Considering both contexts together would allow both between- and within-context differences to be considered, including the role of sex specific moderators^55^.

Finally, formal quantifying and modelling of how perceived risk changes depending on partner number, frequency, and timing of sexual encounters, as well as their subsequent impacts on fitness, would enhance our understanding of how sexual history information is integrated and used to inform partner assessment and mate choice. Such research could also consider contextual factors around past partners, such as deviation from monogamy norms (e.g., extra-pair sex, concurrent sexual partners, or consensual non-monogamy)^14,54^.

## Conclusion

Modern humans are descended from ancestors who effectively navigated the costs, benefits, and risks of sex and relationships. We inherited from them a mating psychology evolved to evaluate the sexual history of potential partners which can acts as a cue of their relationship desires, sexual health, and the presence of past partners who may act as intrasexual competitors. Two aspects of sexual history used to assess a partner’s suitability are their total number of sexual partners and the frequency distribution of their new sexual encounters. Participants of both sexes from all countries examined seemed to use these aspects to inform their decision making – particularly if they were inclined towards long-term mating. While past partner number effects are large, they are integrated with other sexual history information in a nuanced way. Assessment of sexual history is not simply about ‘who’ or ‘what’, but a consideration of ‘how’, ‘why’ and ‘when.’

## Methods

### Participants and procedure

Overall, 5,331 participants were recruited across three studies. In S1, 347 heterosexual adults were recruited online via social media and snowball sampling. In S2, the same sampling methods were used to recruit 467 adults of any sexual orientation who were either of Greek or British Nationality. Greece was selected as a comparison country on an opportunity basis. Some of the research assistants were Greek nationals with access to social networks in Greece. S3 involved an international collaboration of 10 labs which recruited 4,517 adults of any sexual orientation using a variety of recruitment methods (see Table 1). We also attempted to gather data from India via Amazon MTurk for S3, but this resulted in exceptionally poor-quality data (i.e., random, inconsistent responses) which we ultimately excluded. In hindsight, we realised that such issue might not be isolated, as has been reported elsewhere^56,57^.

The procedure was similar in each study. After giving informed consent, participants completed a demographic form and measures of sociosexuality. Next, they completed all three conditions of the sexual history task before receiving an online debrief form.

**Table 1:** Sampling and demographics for studies 1 to 3.

Lab Location	*n*	Targeted countries	Recruitment methods	Survey language	Female (%)	Mean age (SD)	Ethnicity (%)	Sexual orientation (%)	Single (%)
Study 1									
UK	347	None	GP, SM, NC	English	74 (21.3%)	35.6 (10.7)	White: 291 (83.9%) Mixed: 20 (5.8%)	Heterosexual: 347 (100.0%)	137 (39.5%)
Study 2									
UK	467	UK Greece	GP, SM, NC	English	258 (55.2%)	23.2 (6.1)	White: 438 (93.8%)	Heterosexual: 369 (79.0%) Homosexual: 61 (13.1%) Bisexual: 36 (7.7%)	252 (54.0%)
Study 3									
Australia	284	Australia	GP, RC, FC	English	141 (49.6%)	30.5 (8.4)	White: 179 (63.0%) East Asian: 34 (12.0%) South-east Asian: 27 (9.5%) South Asian: 22 (7.8%)	Heterosexual: 212 (74.6%) Bisexual: 45 (15.8%) Homosexual: 17 (6.0%)	143 (50.4%)
Brazil	995	Brazil	GP, SM, NC	Brazilian Portuguese	696 (69.9%)	31.1 (10.5)	White: 651 (65.4%) Mixed (Pardo): 237 (23.8%) Black: 58 (5.8%)	Heterosexual: 691 (69.4%) Bisexual: 183 (18.4%) Homosexual: 93 (9.4%)	541 (54.4%)
China (Mainland)	541	China	GP, RC, FC	Chinese	253 (46.8%)	27.1 (5.0)	East Asian: 541 (100.0%)	Heterosexual: 476 (88.0%) Homosexual: 34 (6.3%)	146 (27.0%)
Czechia	147	Czechia	GP, SM, NC SP, UC, NC	Czech	125 (85.0%)	26.8 (7.2)	White: 146 (99.3%)	Heterosexual: 127 (86.4%) Bisexual: 13 (8.8%)	47 (32.0%)
Greece	262	Greece	GP, SM, NC	Greek	186 (71.0%)	28.1 (9.5)	White: 253 (96.6%)	Heterosexual: 222 (84.7%) Bisexual: 27 (10.3%)	142 (54.2%)
Italy	175	Italy	SP, UC, NC	Italian	100 (57.1%)	29.7 (10.3)	White: 164 (93.7%)	Heterosexual: 137 (78.3%) Bisexual: 27 (15.4%)	76 (43.4%)
China (Macau)	129	China	SP, UC, CC	English	91 (70.5%)	20.1 (1.5)	East Asian: 124 (96.1%)	Heterosexual: 94 (72.9%) Bisexual: 21 (16.3%)	104 (80.6%)
Norway	383	Norway	SP, UC, NC	Norwegian	219 (57.2%)	20.9 (2.3)	Not collected	Heterosexual: 341 (89.0%) Bisexual: 27 (7.0%)	257 (67.1%)
Poland	279	Poland	GP, RC, FC	Polish	141 (50.5%)	25.2 (5.0)	White: 271 (97.1%)	Heterosexual: 238 (85.3%) Bisexual: 30 (10.8%)	111 (39.8%)
UK	1322	China Italy Slovakia UK USA	GP, RC, FC GP, RC, FC GP, SM, NC GP, SM, NC GP, SM, NC	English Italian Slovakian English English	709 (53.6%)	29.8 (11.0)	White: 1018 (77.0%) Southeast Asian: 95 (7.2%) East Asian: 85 (6.4%)	Heterosexual: 1159 (87.7%) Bisexual: 104 (7.9%)	602 (45.5%)

*GP* General population, *SP* Student population, *SM* Social media, *RC* Recruitment company, *UC* University communication, *NC* No compensation, *CC* Course credit, *FC* Financial compensation.

For ethnicity and sexual orientation, only categories representing more than 5% of the sample are displayed.

### Materials

#### Demographics form

This recorded background information including age, sex, ethnicity, sexual orientation, education level, socioeconomic status, and relationship status. In S2 and S3 participants were asked about their country of residence and in which country they spent the most time as a child.

#### Sociosexuality

The revised version of the Sociosexual Orientation Inventory - Revised (SOI-R)^13^ was used to measure sociosexuality. The questionnaire is formed from nine items related to sociosexual behaviour, attitude, and desire measured on nine-point scales. Higher scores indicate a more unrestricted sociosexuality (α > 0.82 in all studies).

#### Sexual history task

In this task, participants respond to images representing different sexual histories. Each image consists of a series of vertical lines, arranged from left to right, with each line reflecting a past sexual partner (see examples in Fig. 1). Images vary along two dimensions. The first is total number of partners (either 4, 12, or 36), chosen based on previous work which found that these attracted high, medium, and low willingness to date ratings respectively^21^. The second is frequency change which has 15 conditions. At one extreme (1) the frequency of new partners sharply increases over time, with the gaps between partners shrinking by 70% from sexual debut onwards. At the other extreme (15), the frequency of sexual partners sharply decreases over time, with the gaps between partners increasing by 70% from sexual debut onwards. The distributions in between show progressively softer increases and decreases respectively (60% for points 2 and 14, 50% for points 3 and 13 and so on) towards the middle distribution (8) where the gaps between partners are equal throughout sexual history. Modifiers above 70% produce distributions with almost no variation across sexual history. The position of the first and last lines are fixed across all images. There are 45 images in total, generated using a custom PHP script and saved as images using screen capture software, allowing insertion into questionnaires.

In the survey itself, participants were told that the leftmost line indicated a suitor’s sexual debut while the rightmost line represented their most recent sexual partner, with whom they had sex six months ago. For each distribution, participants were asked *‘How willing would you be to get involved in a long-term*,* committed relationship with someone whose full sexual history looked like:’* with responses registered on Likert scales spanning from 1 (*Very Unwilling*) to 9 (*Very Willing*). Participants completed the conditions in ascending order of total partners and within each they responded to the distribution showing the sharpest increase in frequency first and the sharpest decrease in frequency last.

### Data analysis approach

All studies used linear multi-level modelling (*lmer* package in R) with participant ID as a random intercept to account for repeated measures. In S2 and S3, we allowed the intercept to vary by participants nested within countries. More complex random effects structures (e.g., random slopes for conditions) could not be implemented due to convergence issues.

For each study, we first modelled the fixed effects of participant sex (sex), past partner number (PPN; 4, 12, and 36), and country (in S2 and S3), as well as their interactions, to examine the effect of PPN on willingness collapsed across frequency distribution (distribution). We then added distribution and its interactions to the model. To examine non-linear effects of distribution, it was included as a continuous variable in its linear, quadratic, and cubic forms using the *poly* function. In follow-up analyses, we determined the non-linear nature of each relationship by identifying the highest order form that was statistically significant.

To examine the impact of sociosexuality, we ran a version of the model on the S3 sample with SOI-R substituted for sex. (Sociosexuality and sex are highly confounded; the inclusion of both could lead to Everest regression effects.). We also ran additional analyses to quantify and compare sociosexuality effects across all studies and samples, examining the 12 PPN condition both for brevity and to reduce the influence of potential ceiling and floor effects. These linear models used (a) average ratings across all distributions to produce a global willingness score and (b) the difference between willingness at the distribution extremes (sharp decrease vs. sharp increase in frequency of new partners over time) to produce a change score.

In all models, we controlled for means-centred age and singlehood status (0 = no, 1 = yes). In S2 and S3, sexual orientation was also included as a series of categorical covariates. Follow-up comparisons were conducted with using the *emmeans* package to examine group differences and slope comparisons. Bonferroni-Holm adjustments were made to *p*-values in post-hoc analysis to reduce the likelihood of Type I errors.

### Country classification

In S3, participants were recruited from labs based in 10 countries. Participants tended to have a nationality congruent with their country of recruitment, but because there were some cases of incongruency (e.g., international students) we sorted them into countries based on where they spent most of their childhood. We then retained countries which had good representation (*n* > 100 participants) and created an ‘other’ category for remaining participants (*n* = 329) to avoid excluding them. We did test an alternative approach whereby participants were sorted into “regions” rather than countries, but this ended up with considerable imbalance within each. The “Oceania” region, for example, had 1 participant from Fiji, 5 from New Zealand, and 230 from Australia.

## Electronic supplementary material

Below is the link to the electronic supplementary material.

Supplementary Material 1

## Data Availability

Data is provided in the supplementary information files accessible here: https://osf.io/b4e8u/
