# Author Correction: Sexual partner number and distribution over time affect long-term partner evaluation: evidence from 11 countries across 5 continents

**DOI:** 10.1038/s41598-025-17764-x

**Published:** 2025-09-12

**Authors:** Andrew G. Thomas, William Costello, Mons Bendixen, Leif Edward Ottesen Kennair, Menelaos Apostolou, Klára Bártová, Ondřej Burýšek, Rob Lowe, Peter Jonason, Marta Kowal, Yago Luksevicius de Moraes, Jiaqing O, Piotr Sorokowski, Danielle Sulikowski, Zuzana Štěrbová, Jaroslava Varella Valentova, Marco Antonio Correa Varella, Yan Wang, Arnaud Wisman, Paula Wright, Steve Stewart-Williams

**Affiliations:** 1 School of Psychology, Swansea University, Swansea, UK; 2 Department of Psychology, University of Texas at Austin, Austin, USA; 3 Department of Psychology, Norwegian University of Science and Technology, Trondheim, Norway; 4 Department of Social Sciences, University of Nicosia, Nicosia, Cyprus; 5 Department of Psychology and Life Sciences, Faculty of Humanities, Charles University, Pátkova, Czechia; 6 Psychology Research Institute, University of Economics and Human Sciences, Warsaw, Poland; 7 IDN Being Human Lab-Institute of Psychology, University of Wrocław, Wroclaw, Poland; 8 Department of Psychology, Faculty of Philosophy, Fundação Santo André, Santo André, Brazil; 9 Department of Experimental Psychology, Institute of Psychology, University of São Paulo, São Paulo, Brazil; 10 Department of Psychology, University of Macau, Macau, China; 11 Department of Psychology, Aberystwyth University, Aberystwyth, UK; 12 Institute of Psychology, University of Wrocław, Wrocław, Poland; 13 School of Psychology, Charles Sturt University, Wagga Wagga, Australia; 14 Department of Psychology, Faculty of Arts, Charles University, Prague, Czechia; 15 Department of Experimental Psychology, Institute of Psychology, University of Sao Paulo, Sao Paulo, Brazil; 16 Department of Psychology, Fudan University, Shanghai, China; 17 School of Psychology, University of Kent, Canterbury, UK; 18 Department of Life Sciences, Brunel University London, Uxbridge, UK; 19 School of Psychology, University of Nottingham Malaysia, Semenyih, Malaysia

Correction to: *Scientific Reports* 10.1038/s41598-025-12607-1, published online 18 July 2025

The original version of this Article contained an error in the spelling of the author Jiaqing O, which was incorrectly given as O. Jiaqing.

Additionally, in Table 1, first column headings were misaligned and shifted by one,

The correct and incorrect Table 1 appear below.

Incorrect:

**Table:**
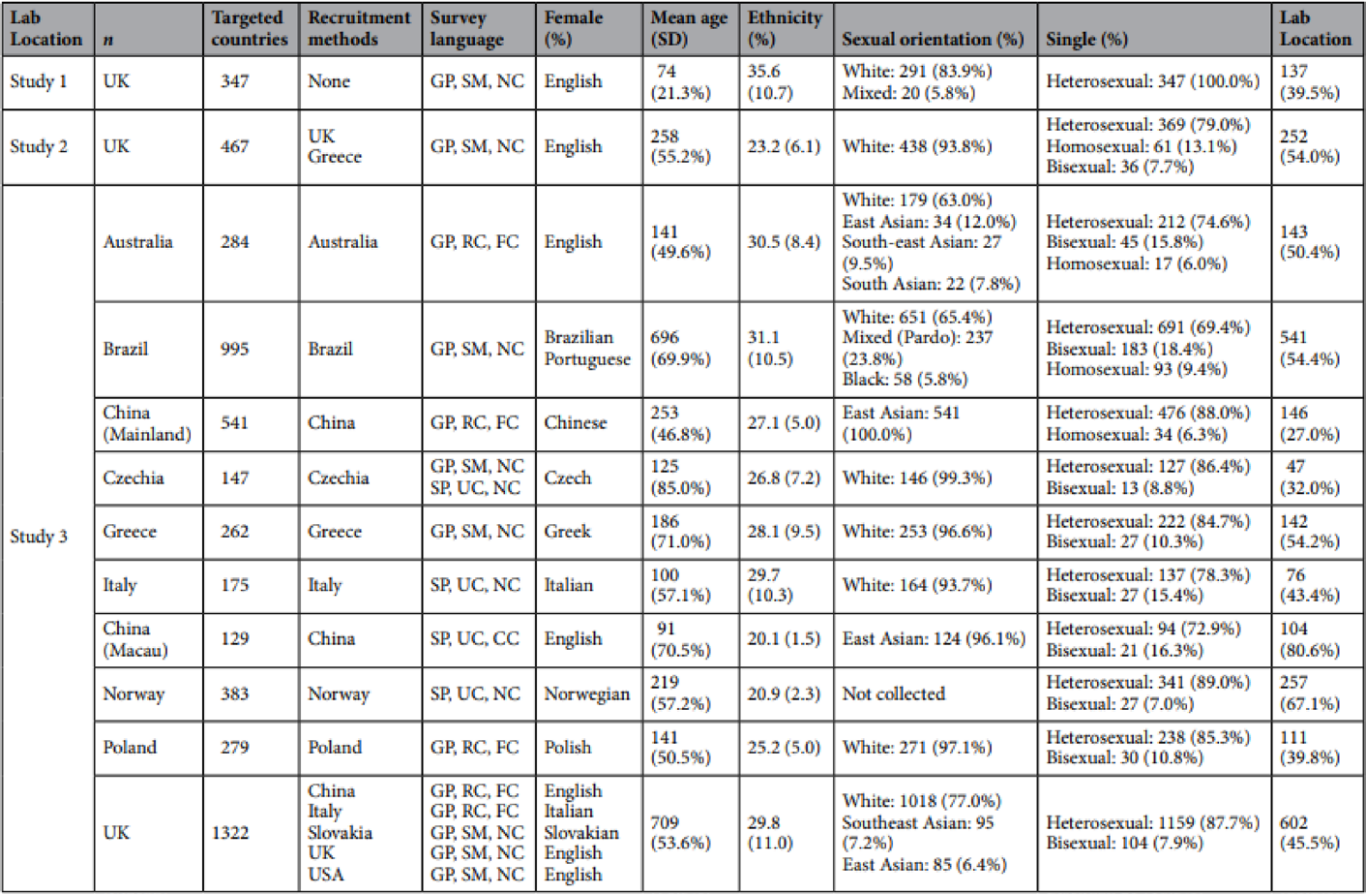

Correct:

**Table 1:** Sampling and demographics for Studies 1 to 3.

Lab Location		*n*	Targeted countries	Recruitment methods	Survey language	Female (%)	Mean age (*SD*)	Ethnicity (%)	Sexual Orientation (%)	Single (%)
Study 1										
	UK	347	None	GP, SM, NC	English	74 (21.3%)	35.6 (10.7)	White: 291 (83.9%) Mixed: 20 (5.8%)	Heterosexual: 347 (100.0%)	137 (39.5%)
Study 2										
	UK	467	UK Greece	GP, SM, NC	English	258 (55.2%)	23.2 (6.1)	White: 438 (93.8%)	Heterosexual: 369 (79.0%) Homosexual: 61 (13.1%) Bisexual: 36 (7.7%)	252 (54.0%)
Study 3										
	Australia	284	Australia	GP, RC, FC	English	141 (49.6%)	30.5 (8.4)	White: 179 (63.0%) East Asian: 34 (12.0%) South-east Asian: 27 (9.5%) South Asian: 22 (7.8%)	Heterosexual: 212 (74.6%) Bisexual: 45 (15.8%) Homosexual: 17 (6.0%)	143 (50.4%)
	Brazil	995	Brazil	GP, SM, NC	Brazilian Portuguese	696 (69.9%)	31.1 (10.5)	White: 651 (65.4%) Mixed (Pardo): 237 (23.8%) Black: 58 (5.8%)	Heterosexual: 691 (69.4%) Bisexual: 183 (18.4%) Homosexual: 93 (9.4%)	541 (54.4%)
	China (Mainland)	541	China	GP, RC, FC	Chinese	253 (46.8%)	27.1 (5.0)	East Asian: 541 (100.0%)	Heterosexual: 476 (88.0%) Homosexual: 34 (6.3%)	146 (27.0%)
	Czechia	147	Czechia	GP, SM, NC SP, UC, NC	Czech	125 (85.0%)	26.8 (7.2)	White: 146 (99.3%)	Heterosexual: 127 (86.4%) Bisexual: 13 (8.8%)	47 (32.0%)
	Greece	262	Greece	GP, SM, NC	Greek	186 (71.0%)	28.1 (9.5)	White: 253 (96.6%)	Heterosexual: 222 (84.7%) Bisexual: 27 (10.3%)	142 (54.2%)
	Italy	175	Italy	SP, UC, NC	Italian	100 (57.1%)	29.7 (10.3)	White: 164 (93.7%)	Heterosexual: 137 (78.3%) Bisexual: 27 (15.4%)	76 (43.4%)
	China (Macau)	129	China	SP, UC, CC	English	91 (70.5%)	20.1 (1.5)	East Asian: 124 (96.1%)	Heterosexual: 94 (72.9%) Bisexual: 21 (16.3%)	104 (80.6%)
	Norway	383	Norway	SP, UC, NC	Norwegian	219 (57.2%)	20.9 (2.3)	Not collected	Heterosexual: 341 (89.0%) Bisexual: 27 (7.0%)	257 (67.1%)
	Poland	279	Poland	GP, RC, FC	Polish	141 (50.5%)	25.2 (5.0)	White: 271 (97.1%)	Heterosexual: 238 (85.3%) Bisexual: 30 (10.8%)	111 (39.8%)
	UK	1322	China Italy Slovakia UK USA	GP, RC, FC GP, RC, FC GP, SM, NC GP, SM, NC GP, SM, NC	English Italian Slovakian English English	709 (53.6%)	29.8 (11.0)	White: 1018 (77.0%) Southeast Asian: 95 (7.2%) East Asian: 85 (6.4%)	Heterosexual: 1159 (87.7%) Bisexual: 104 (7.9%)	602 (45.5%)

*Note*: GP = General population; SP = Student population; SM = Social media; RC = Recruitment company; UC = University communication; NC = No compensation; CC = Course credit; FC = Financial compensation. For ethnicity and sexual orientation, only categories representing more than 5% of the sample are displayed.

The original Article has been corrected.

